# Long-acting injectable depot buprenorphine from a harm reduction perspective in patients with ongoing substance use and multiple psychiatric comorbidities: a qualitative interview study

**DOI:** 10.1186/s12954-024-00984-1

**Published:** 2024-03-25

**Authors:** Björn Johnson, Bodil Monwell, Andrea Johansson Capusan

**Affiliations:** 1 School of Social Work, Lund University, Lund, Sweden; 2 Department of Psychiatry, County Hospital Jönköping, Jönköping, Sweden; 3 Department of Social Work, Jönköping University, Jönköping, Sweden; 4 Department of Psychiatry in Linköping, Department of Biomedical and Clinical Sciences, Linköping University, Linköping, Sweden; 5 Center for Social and Affective Neuroscience, Department of Biomedical and Clinical Sciences, Linköping University, Linköping, Sweden

**Keywords:** Opioid use disorder, Treatment, Polydrug use, Psychiatric comorbidity, Long-acting injectable depot buprenorphine, Qualitative interviews.

## Abstract

**Background:**

Long-acting injectable depot buprenorphine may increase access to opioid agonist treatment (OAT) for patients with opioid use disorder in different treatment phases. The aim of this study was to explore the experiences of depot buprenorphine among Swedish patients with ongoing substance use and multiple psychiatric comorbidities.

**Method:**

Semi-structured qualitative interviews were conducted with OAT patients with experience of depot buprenorphine. Recruitment took place at two OAT clinics with a harm reduction focus, specializing in the treatment of patients with ongoing substance use and multiple comorbidities. Nineteen participants were included, 12 men and seven women, with a mean age of 41 years (range 24–56 years), and a mean of 21 years (5–35 years) of experience with illicit substance use. All participants had ongoing substance use and psychiatric comorbidities such as ADHD, anxiety, mood, psychotic and eating disorders. Interviews were transcribed verbatim. Thematic content analysis was conducted both manually and using qualitative data analysis software.

**Results:**

Participants reported social benefits and positive changes in self-perception and identity. In particular, depot buprenorphine contributed to a realization that it was possible to make life changes and engage in activities not related to substance use. Another positive aspect that emerged from the interviews was a noticeable relief from perceived pressure to divert OAT medication, while some expressed the lack of income from diverted oral/sublingual OAT medication as a negative, but still acceptable, consequence of the depot buprenorphine. Many participants considered that the information provided prior to starting depot buprenorphine was insufficient. Also, not all patients found depot buprenorphine suitable, and those who experienced coercion exhibited particularly negative attitudes towards the medication.

**Conclusions:**

OAT patients with ongoing substance use and multiple psychiatric comorbidities reported clear benefits of depot buprenorphine, including changes in self-perception which has been theorized to play an important role in recovery. Clinicians should consider the specific information needs of this population and the extensive diversion of traditional OAT medications in this population to improve the treatment experience and outcomes. Overall, depot buprenorphine is a valuable treatment option for a population in need of harm reduction and may also contribute to psychological changes that may facilitate recovery in those with the greatest need.

**Supplementary Information:**

The online version contains supplementary material available at 10.1186/s12954-024-00984-1.

## Background

Novel treatment options with long-acting injectable depot buprenorphine (hereafter: depot buprenorphine) for opioid use disorder have increased access to opioid agonist treatment (OAT) in recent years. In pivotal trials, depot injections have shown similar efficacy to sublingual buprenorphine/naloxone [1] and superior efficacy to placebo [2]. The first depot injection was approved in the USA in 2017 [3] and since 2019, weekly [1] and/or monthly [1, 2] subcutaneous buprenorphine injections are available for the treatment of moderate to severe opioid use disorder (OUD) in the EU, UK, Canada, Australia, New Zealand and several countries in the Middle East and North Africa. Patient-reported outcomes from RCTs and observational studies indicate long-term safety and increased patient satisfaction with depot injections [4, 5].

### Patient perspectives on depot buprenorphine

Interestingly, the introduction of depot buprenorphine has also led to a renewed research interest in patient perceptions and attitudes towards OAT in general and depot buprenorphine in particular.In recent years, several studies have investigated how depot buprenorphine affects patients’ lives, their treatment, and their relationships with treatment staff [6–9]. Our research group conducted a stratified qualitative interview study [7] to explore reasons for choosing depot buprenorphine and reasons for discontinuing or declining this treatment. Qualitative studies describe benefits in terms of practical factors, an increased sense of freedom, psychological benefits such as a reshaping of self-identity to feel “normal” [6, 7], and a reduction in the stigma associated with daily supervised OAT [8]. Loss of contact with staff, the need for a daily routine, and concerns about medication effects and side effects are reasons for choosing to discontinue depot buprenorphine or continue with sublingual treatment [9]. A trusting relationship with treatment staff and adequate information are important for successful induction of depot buprenorphine [7]. In contrast, mistrust and coercion could lead to a “polluted pharmaceutical atmosphere”, similar to that described during the clinical introduction of sublingual buprenorphine/naloxone combinations, which negatively affected patients’ perceptions of medication effects and side effects [10].

In the early stages of clinical implementation, depot buprenorphine was predominantly offered to more stable patients, which explains why early qualitative studies [6–8] did not capture how depot buprenorphine affected patients at the most severe end of the OUD spectrum with ongoing polysubstance use and multiple comorbidities. This perspective is important for the use of buprenorphine depot injections. Firstly, research from other medical fields such as the treatment of schizophrenia, with decades of experience in depot injections, suggests that the most unstable patients may benefit most from these formulations [11]. In addition, unstable patients may face several barriers to accessing care, including poor treatment adherence and clinicians’ concerns that treatment may be harmful [12, 13] which must be balanced against the considerable harms of untreated OUD. It is therefore important to explore how unstable patients themselves experience the potential benefits and disadvantages associated with depot buprenorphine.

### The setting

In Sweden, national regulations require OAT to be provided in a specialised psychiatric or addiction care setting, registered with the Health and Social Care Inspectorate [14, 15]. The treatment must include both medication and, for those patients who need it, psychological or psychosocial treatment and support, either provided by the unit or in collaboration with the municipal social services or other care providers [15].

The availability of OAT is low compared to many Western European countries. The contrast with neighbouring Denmark and Norway, with more than twice as many OAT patients per 100 000 inhabitants than Sweden, is stark [16, 17]. Access to treatment also varies considerably across the country [16]. There are no current estimates of the prevalence of opioid dependence in the Swedish population, but drug-related deaths increased steadily over the period 2000–2017 and are among the highest in Europe [18]. However, there has been a slight decrease in deaths since 2017, in parallel with an expansion of harm reduction interventions such as naloxone distribution, needle exchange programs and an increased access to OAT.

The number of OAT patients in Sweden has increased continuously over the last decades, from about 1000 patients in 2000 to about 7500 patients in 2022 [16]. The most common OAT medication is still methadone, followed by sublingual buprenorphine and sublingual buprenorphine-naloxone combination. Since 2007, the Swedish National Board of Health and Welfare has recommended buprenorphine-naloxone as the first-line medication in OAT, but in many local settings, implementation of these recommendations has been hampered by local traditions and patient resistance. Depot buprenorphine was introduced in 2019. In 2022 about 10% of Swedish OAT patients were prescribed depot formulations.

For many years OAT was a controversial treatment modality in Sweden. Access was strictly regulated, with high thresholds for entry, a strong focus on abstinence and rehabilitation [19], and an emphasis on the potential harms caused by the treatment itself, while disregarding the harms of untreated OUD. Harm reduction-oriented OAT did not exist, and patients with repeated relapses into illicit substance use were discharged from treatment according to earlier national guidelines [20]. This led, among other things, to the emergence of a significant illicit market for OAT medicines [21].

Since 2015, however, the national regulatory framework for OAT has been brought in line with modern research, with lower thresholds for entering treatment [19] and no longer recommending involuntary discharge, while emphasizing harm reduction measures such as naloxone distribution [22]. The metropolitan areas now have clinics with a strong harm-reduction profile, with units specialising in clients with ongoing substance use and multiple somatic and psychiatric co-morbidities. However, there are considerable variations across the country, and in some healthcare regions involuntary discharge is still a common practice [16].

Swedish OAT has traditionally had a strong focus on control and medical safety. According to the current national regulations, which have not yet been adapted to the depot formulations, the prescribed medication must be taken daily under clinic supervision for the first three months. After that, if the treatment outcome is stable, the doctor can gradually allow the patient to manage their own medication [23, 24].

### The study

In this study, we aimed to investigate the experiences of patients with ongoing, severe substance use and multiple comorbidities who are receiving long-acting injectable depot buprenorphine within a harm reduction setting in Sweden. Our goal was to generate new insights into the potential benefits and challenges associated with depot buprenorphine in this specific patient population. Specifically, we explored how the adoption of depot buprenorphine impacts various aspects of patients’ lives, including their treatment experiences, relationships with treatment staff, and perspectives on their future opioid agonist treatment (OAT). By focusing on this subgroup, we aimed to contribute valuable knowledge to enhance the understanding and optimization of depot buprenorphine use in the context of harm reduction for patients with complex needs.

## Methods

The study is based on qualitative, semi-structured interviews with OAT patients with ongoing substance use and multiple comorbidities.

### Sampling and recruitment

Participants were recruited between December 2021 and May 2022 from two harm reduction units specializing in the treatment of patients with ongoing, severe polysubstance use and multiple comorbidities. The units were part of two larger OAT clinics, each serving approximately 600–700 patients, located in two large cities (> 300,000 inhabitants). In the first clinic, around 100 patients, of whom just under half were treated with depot buprenorphine, had contact with the harm reduction unit. In the second clinic, about 150 patients had contact with the harm reduction unit, but only about ten of them were treated with depot buprenorphine. The treatment staff included doctors specialized in psychiatry or addiction medicine, nurses, and mental health workers. Additionally, the clinics could provide access to medical workups and hepatitis C treatment, as well as services from psychologists and occupational therapists.

Inclusion criteria for the study were having (1) OAT at one of the two units with harm reduction profile included in the study, (2) ongoing substance use and (3) multiple comorbidities (such as, but not limited to, substance use disorders other than OUD, psychiatric disorders including affective, anxiety or psychotic disorder, somatic comorbidities including hepatitis C); and (4) willingness to participate. Exclusion criteria were the inability to give informed consent, either because of poor language skills or because they were too impaired by substances and/or mental illness at the time of the information. Excluded participants were given the opportunity to return at a later date if they wished to participate in the study.

Posters, flyers and information to clinical staff were used to inform patients who were part of the target group about the opportunity to participate. Interested patients were given a date and time for the interview, which usually coincided with a visit for their depot injection or other medication collection. As patients with ongoing substance use and/or mental illness may find it difficult to attend scheduled appointments, the interviewer was also available in the reception area of the clinics, without prior booking, allowing for the opportunity to meet with respondents who missed their scheduled appointments.

All interviews were conducted by BM, a clinical researcher with extensive experience in conducting qualitative interviews with patients who use psychoactive substances and have severe psychiatric comorbidities. BM had previously worked as healthcare counsellor at another OAT clinic but had no previous relationship with the clinics or patients in question. Recruitment and interviews continued until BM deemed that data saturation had been achieved.

### Ethical considerations

Interviewing patients with active use and comorbidities poses several ethical challenges. Patients may be too affected by substances or by mental illness to give informed consent or to participate in the interview at a given time. This could limit the possibility of the most vulnerable patients to have their voices heard. We chose to handle this dilemma by conducting an individual clinical assessment of potential participants prior to the interviews. BM conducted a clinical assessment to evaluate the participants’ degree of influence of psychoactive substances, potential cognitive impairment, and current psychiatric status. Assessments were recorded in field notes.

Another aspect was that many patients had experience of involuntary discharge and it was important to ensure confidentiality. Participants received both verbal and written information about the study. They were informed that the interviews would be confidential, that they could discontinue the interview at any time, that all data would be pseudonymized before publication, and that their participation would not affect their treatment in any way. Subsequently, the patients signed an informed consent form. In the case of patients interviewed by telephone, staff at the clinic provided written information and obtained written consent before the interview. Participant characteristics are reported only at the group level, to avoid the risk of individual participants being identified.

The study was reviewed and approved by the Swedish Ethical Review Authority (Ref. No. 2020 − 00796).

### Interview procedure

Semi-structured interviews were conducted using an interview guide covering the following themes: (a) background and history of substance use, (b) previous treatment experiences, (c) experiences with and views on OAT, (d) relationships with treatment staff, (e) views on control and support in ongoing treatment, (f) thoughts on the choice of drug formulation, (g) perceptions of the information provided by staff about depot buprenorphine, and (h) thoughts about the future.

The data consist of nineteen interviews. Eighteen were conducted face-to-face, in a secluded room at the clinic in question, and one was conducted by telephone [7, 25]. Seven participants were perceived to be affected by substance intake (six of them confirmed this) and a further two were experiencing withdrawal symptoms. None of them were disoriented or considered so substance-impaired that they were unable to give informed consent or that it would be impossible to conduct an interview. However, four other people were excluded from the study: three did not meet the inclusion criteria (two were stable and in remission, one did not speak sufficient Swedish) and one was excluded due to agitated, aggressive behavior at the clinic. After the interviews, the participants received a shopping voucher worth SEK 200 (about €20).

The interviews lasted on average 37 min (range 21–55 min). They were recorded on a digital voice recorder and then transcribed verbatim by BM.

### Analysis

Thematic analysis [26] was carried out in two ways. The material was thoroughly read and coded by BM and AJC based on the themes outlined in the interview guide. This was followed by a detailed coding, in which different patterns in the interview responses were identified. The themes and sub-themes were then compiled in an Excel spreadsheet. In parallel, and blinded to the above findings, BJ conducted a computer-assisted thematic analysis using NVivo (Release 1.7, QSR International 2022). Initially, an inductive coding was carried out manually in NVivo. This coding was then carefully reviewed, with some codes modified and others merged. Subsequently, more general categories and subcategories were created. The parallel categorizations in Excel and NVivo were then compared and found to be largely consistent. In the final step of the analysis, the categories and codes were reviewed once more in NVivo and illustrative quotes were selected from the relevant text passages. The selection was made by BJ, who also wrote the first draft of the results section. The quotes were translated into English using ChatGPT 3.5 and then proofread by a native English translator.

## Results

### The participants

Nineteen participants, 12 men and 7 women, mean age 41 years (range 24–56 years) were included. Eighteen had ongoing depot buprenorphine treatment, 12 with weekly injections and 5 with monthly injections. One participant had recently discontinued depot buprenorphine treatment and switched back to sublingual mono-buprenorphine.

Study participants had a diagnosed opioid use disorder and extensive experience of polydrug use. The mean duration of illicit drug use was 21 years (5–35 years). Before starting OAT, nine participants had used heroin as their primary drug, two had mainly used other opioids, and six had switched between heroin and other opioids. Two reported primary drugs other than opioids. Many had started using drug in their early teens and spoke of a childhood spent in difficult circumstances, including parents with drug problems, lack of care, and traumatic experiences.

All participants had received some treatment prior to starting OAT. Nine had extensive treatment experience for substance use disorders, including compulsory treatment, while the rest had more limited treatment experience prior to OAT. Most had been in OAT for a relatively short time, three years or less, but some had long experience and had been involuntarily discharged from OAT several times. As mentioned above, involuntary discharge used to be common practice in Sweden.

As the interviews were conducted in clinics for unstable patients, almost all participants had ongoing illicit substance use. At the time of the interview, nine participants reported extensive use, eight had more limited use, and one reported short-time abstinence, of less than three months. In one case, the participant did not provide information on drug status. There was a high level of psychiatric comorbidity in the group, with ADHD or other neurodevelopmental disorders, current or lifetime anxiety disorders, mood disorders, trauma and/or psychosis. Experiences of eating disorders and intentional self-harm were also reported.

### Freedom of choice and information

Participants were offered depot injections due to their ongoing instability, with the intention of improving adherence and/or reducing the risk of overdose. Several patients reported that they had been advised by staff to try depot buprenorphine, because of inadequate effectiveness or side effects from previous medication. Some had asked to try depot buprenorphine themselves, having been recommended to do so by peers or having otherwise come across positive information. Most participants reported that the decision to try depot buprenorphine was voluntary. However, some reported feeling that the staff had effectively forced them to choose between depot buprenorphine and involuntary discharge from OAT, either because they were suspected of selling medication, or because they missed scheduled appointments or otherwise mismanaged their treatment. One participant reported being presented with a fait accompli:

*“When I came [to the clinic] before Christmas, there was only one bottle and one syringe here when I was supposed to get my dose. […] They just said I would get this [a depot injection] instead, something about it being Christmas and New Year and they couldn’t dispense tablets daily, so I got this instead. They said it was the same dose as the tablets.” (Male participant #3)*.

In addition to exploring participants’ opinions about voluntariness, we also inquired about the adequacy of the information they had received before starting depot buprenorphine treatment. While some participants felt that they had been adequately informed, others had gained knowledge by observing friends or partners who had tried depot buprenorphine. Nevertheless, a significant number of participants, including some who were enthusiastic about depot buprenorphine, reported that they had not received enough information. For instance, one participant stated:

*“No, I didn’t get so much information other than that it [the dose] would last for a week. That it was in injection form, I wouldn’t have to come every day. And that it works just like usual.” (Female participant #6)*.

### Effects and side effects of depot buprenorphine

The perceived effects and side effects of the medication were a topic that was addressed in all interviews. A clear majority of the participants reported being satisfied with depot buprenorphine and described the depot effect as more even and stable than treatment with sublingual tablets.

*“On Subs [buprenorphine tablets], I felt worse in the evenings and in the morning, when you wake up and so on. It feels like the “sub” wears off when you sleep. Then it takes a while before it starts working again. You dip quickly. Now it’s even.” (Male participant #14)*.

Participants described feeling good, harmonious, and/or more “normal”. Some who had previously supplemented their medication with heroin or other illicit opioids, reported that they no longer needed to do so. The craving was gone, and so were the thoughts of heroin.

*“I have no craving for heroin anymore, it’s completely insane. Because I had it on the tablets all the time, for all those years. I had to work with the craving all the time. But the depot buprenorphine, they kind of just cut it off.” (Female participant #19)*.

Some participants said that they felt that the medication was right for them from the start, while others reported that the effect had varied during the first few weeks. The latter reported decreasing effects and withdrawal symptoms that became noticeable or significant towards the end of the week. Some said that they sometimes bought illicit buprenorphine to balance the effect. During the titration phase, many patients reported being offered earlier refills or extra doses in the form of sublingual tablets to counter withdrawal symptoms.

*“I think it’s going great. Except on the weekends… I have it weekly, so on Saturday afternoon it starts to run out. You get cold sweats. (…) I have to run out to buy Subs on the street. I just have to.” (#15, Woman, 56 years)*.

Use of illicit substances – mainly benzodiazepines or other sedatives or hypnotics – was common among the patients but notably the participants did not relate this to the depot buprenorphine. Instead, they described it as something they chose to do because they enjoyed it, or to cope with anxiety and poor mental health, or as a habit they had had for a long time and did not think they could stop. However, most participants reported that their use of illicit substances decreased when they started with the depot buprenorphine.

There were also some participants who were dissatisfied with the depot buprenorphine. They reported that the medication was not effective enough against drug cravings, or that the effect wore off after a few days, resulting in cravings and gradually increasing withdrawal symptoms. All of these people had an extensive use of illicit substances, which they described as a way of boosting their medication. Several of the dissatisfied participants stated that they had been negative towards depot buprenorphine from the beginning and had felt coerced by the staff. One person had switched back to sublingual tablets and two others said that they wanted to do so.

Just over half of the participants reported side effects, mostly described as temporary or mild. Pain, tenderness or “lumps” at the injection site were the most common. Some also described side effects such as tingling, numbness, dry mouth, brief nausea and headache after the injection which they related to temporary too high dose exposure. These side effects resolved over time or after dose adjustment. Several participants mentioned typical opioid-related side effects such as constipation, stomach problems, and sweating, but these problems were described as milder than with sublingual buprenorphine or methadone. Overall, participants described more side effects and negative experiences with other formulations than with depot buprenorphine.

### Social benefits of depot buprenorphine

All the participants who had a positive view of depot buprenorphine talked about various social benefits that the injections had given them. The most commonly reported benefit was that depot buprenorphine meant that the patients no longer had to follow the “traditional” Swedish OAT structure (see background section), which was perceived as time-consuming and/or uncomfortable. Often, this was about avoiding the stress and anxiety that could result from having to get up early and go to the clinic every day.

*“You don’t have to rush and feel anxiety about coming here. Otherwise, you must get up every morning, feel bad [due to early withdrawal symptoms] and take the bus all the way here. Meet a load of people everywhere to get your dose. With all that anxiety the whole time, which starts the night before. Damn. But when I got [the depot buprenorphine], I felt good, was just… healthy all the time… you know, I could wake up at 8 in the morning and feel that everything was fine… and then I could go back to sleep for a while.” (Male participant #3)*.

Avoiding meeting other patients who were under the influence of drugs, or from whom they had other reasons to stay away, was also mentioned as a benefit by several people.

*“Before, when I picked up my [buprenorphine tablets], I picked them up in the afternoon because it affected me a lot to come in the morning and see everything that was going on here… yes, how they [the other patients] are. It’s tough seeing them when they’re under the influence.” (Female participant #22)*.

As well as highlighting what they did not have to do, some participants emphasized the increased freedom that depot buprenorphine gave them – the freedom to travel and see relatives, and to have more control over their own time. *“No, but still, my ambition is to get monthly injections. To get this higher degree of freedom. I think it’s not only beneficial for me, it’s beneficial for all individuals after a time.” (Male participant #7)*.

### Positive changes in self-perception and identity

In addition to the social benefits of depot buprenorphine, many patients also reported more profound changes in perspective and daily life – that depot buprenorphine could help you to *“shift the focus in life […], to self-realization instead of destructiveness” (Male participant #12)* as one participant put it. This type of benefit was described mainly by patients who did not have an extensive use of illicit substances. *“You don’t have to think about the fact that you are, like, a former junkie. You don’t have to think about your life as a drug addict. That’s not what you are, you’re a human being.” (Male participant #12)*.

Participants reported that depot buprenorphine had helped them realize that they could make positive changes in their lives. *“I can do things in my life. I’m not tied down anymore. I’m tied to this place [the clinic], but not in the same way. Not tied to addiction… and not tied to medication either.” (Male participant #7)* Several also mentioned engaging in other activities to fill their day, such as dating, working out, or cooking, after starting depot buprenorphine treatment.

Some participants described the opportunity to shift focus as an almost life-changing transformation of their self-image and identity. They no longer lived as people with addiction, and therefore did not need to identify as such.

*“The biggest lifestyle difference between the tablets and [depot buprenorphine], I think, is that I can feel more like a normal… um… normal person. I don’t have to identify as a… as the addicted person in the same way now that I get the injections (…) This is the person I want to be. The person I am today, who can stand for their decisions and be a good fellow human being. Make good decisions for myself and others.” (Male participant #21)*.

However, changing one’s identity could also be a challenging or even frightening experience. One person, who had experienced severe drug problems since her early teens, described it as a strange feeling to suddenly be able to be someone else, but at the same time not knowing what to do with the rest of one’s life.

*“I have a disability pension. I don’t know if I’ll get a job or something… I don’t have anyone [to talk to]. But I thought I would start talking to my contact person. So maybe I could start with some kind of activity. To pass the time. (…) Because I am alone during the days now. I just sit, sit at home.” (Female participant #6)*.

### Diversion and the illicit buprenorphine market

One of the obvious advantages of depot buprenorphine is that it cannot be diverted, i.e., sold to or shared with people outside of treatment. In the interviews, we asked questions about the illicit buprenorphine market and what depot buprenorphine could mean for this market.

Many patients testified to a relatively extensive illicit trade in buprenorphine tablets associated with OAT programs. There was often *“a damn pestering*” (Male participant #16) from people wanting to buy. *“As soon as I walk out of this door here, if you go to the regular [clinic] and pick up [tablets] during the day, then there are at least thirty people asking to buy.” (Female participant #22)* The costumers are other people with opioid dependence, *“those who have dropped out of [treatment] or who were our friends when we were still using.” (Female participant #15)*.

Several participants said that it was nice to be able to avoid the hassle by referring to receiving depot buprenorphine. In fact, some of them described this as one of the greatest benefits of this treatment. *“It’s great! I’m so happy to be able to say it: ‘You can’t suck out my Subutex because it’s in my arm’” [laughs and taps his arm]. (Male participant #15)* Another participant stated: *“Even today, people who don’t know I’m on depot call me. But it [the medication] is in my stomach, it’s not on the table, I have nothing to sell [chuckles].” (Female participant #6)*.

That patients who receive sublingual tablets often sell part of their dose was a common perception among the patients we interviewed. An eight-milligram tablet can be sold for 150–300 kronor [approx. 13–26 €] in the city where we conducted most of the interviews. Such sales can therefore provide a significant extra income. *“It’s very common. […] If you think about it, three hundred kronor a day… that’s 9,000 [800 €] a month.” (Male participant #13)*.

Several participants suggested that economic motives often played a decisive role for patients who declined or discontinued depot buprenorphine treatment.

*“[They would say], ‘No, this [depot buprenorphine] doesn’t work for me, it’s crap.’ But I think that’s bullshit. 99% of it has to do with either using other substances and wanting to keep the option to do so, or to sell a part of their medication.” (Male participant #21)*.

Participants also shared their own experiences of selling tablets. One person said that he had previously sold a part of his dose for economic reasons, but that he did not regret starting depot buprenorphine treatment.

*“It certainly changed my financial situation a little bit. But based on the stability and well-being I get from [depot buprenorphine], it’s priceless. So [depot buprenorphine], for me, it’s the holy grail. There’s nothing I would choose over [depot buprenorphine], I wouldn’t even choose heroin.” (Male participant #21)*.

## Discussion

In this study we explored the experiences of depot buprenorphine treatment in unstable OAT patients with severe ongoing polysubstance use and multiple psychiatric comorbidities.

While the positive and negative aspects reported by this group of patients were similar to those reported in previous Australian and Swedish studies of more stable OAT patients [6–8], several treatment aspects emerged that were more specific to this treatment group.

It is particularly interesting that this group of unstable patients describe similar positive changes in self-perception and identity as shown in studies with more unselected groups of OAT patients [6, 7]. People with long-term problems with illicit drugs often develop a lifestyle with particular values, skills and livelihoods associated with drug use. Over time, many lose their networks and anchorage in “normal” society and develop an identity as a “deviant” or “outsider” [27–29]. It is in the drug subculture that they are rooted, have most of their social relationships and feel a sense of belonging. Many studies emphasise the importance of changing one’s identity in order to move away from drug use and bring about lasting change [27, 28, 30, 31]. For this to be successful, individuals need to break with their previous lifestyle and resume or establish social relationships outside the drug-using subculture. The stories of the participants in this study suggest that depot buprenorphine can be an important facilitator in such a process of change. It can free up time, allow a change of focus in life, and reduce exposure to people and environments associated with the drug subculture.

Diversion to the illicit market is a well-documented problem in OAT [21, 32] and has been a primary motivation for the development of buprenorphine-naloxone combinations. The practical impossibility of diversion has been emphasized as a major advantage of depot buprenorphine. It is therefore rather surprising that previous studies on depot buprenorphine have not explored patients’ views on this issue. Our study is the first to explicitly examine the importance of the illicit buprenorphine market in patients’ decision-making regarding sublingual versus depot buprenorphine.

As in previous research [21], our interviews revealed a significant illicit market for buprenorphine tablets. Many participants recounted their own experiences of using illicit buprenorphine prior to starting OAT. This use usually took the form of low doses administered intranasally or intravenously, although sublingual use of illicit buprenorphine also occurred. Such use often had pseudo-therapeutic motives, for example when patients had difficulty obtaining or retaining a place in regular treatment [33].

It can be difficult to obtain reliable information about sensitive topics through interviews, particularly in relation to prohibited or stigmatizing behaviors that the participants may have engaged in themselves [21]. However, the participants in this study were unexpectedly candid about diversion, including the negative impact of decreased diversion on their income. Pressure to sell their medication appeared to be part and parcel of their daily lives. Several participants also shared their own experiences of selling medication before starting depot buprenorphine treatment. This is consistent with previous research describing patients with ongoing use, whose social contacts include others with active use, as most likely to engage in diversion [21].

Drug subcultures often develop what the anthropologist Philippe Bourgois [34] has called a “moral economy of sharing”, i.e. a system of norms in which it is considered unethical not to share drugs with friends who are “drug sick”. In this moral economy, economic and altruistic motives often go hand in hand [35, 36]. Breaking with such a norm system is difficult and does not happen automatically simply by starting treatment where different rules are supposed to apply. As noted above, successful disengagement often requires breaking away from your old network in the drug culture and creating a new, drug-free social network. The accounts of participants in this study suggest that depot buprenorphine may facilitate such disengagement.

Although they may continue to use other drugs, these unstable patients clearly experienced reduced opioid craving and increased stability. When treated with sublingual formulations, both missing doses and diversion are common, potentially leading to suboptimal medication levels. The positive effects of depot buprenorphine may in fact reflect unstable patients receiving a sufficient dose of buprenorphine, which is necessary for effective treatment retention [37]. Additionally, depot buprenorphine may increase access to treatment, which is particularly important given that this group of patients may not be offered OAT to the same extent as more stable patients with better adherence [12, 13].

Conversely, insufficient medication effects towards the end of the dose periods (in our population mostly weekly injections) may contribute to relapse and continued substance use among unstable patients, who are close to the illicit market. Insufficient effects were particularly evident during titration, but the problem may persist in some patients. It is important to take patients’ experiences into account and make appropriate dose adjustments or use monthly formulations to increase stability.

Like previous research [7, 9], this study suggests that depot buprenorphine is not suitable for all patients. People who are skeptical before trying depot buprenorphine often remain so, and many in this group seem to discontinue the treatment. It is therefore necessary and appropriate to offer patients a choice of different formulations. New patients and unstable patients can be offered depot buprenorphine or a buprenorphine-naloxone combination as alternatives for buprenorphine treatment. Both earlier findings [21] and the findings of this study indicate that mono-buprenorphine tablets entail a higher risk of diversion.

Changing medications can cause frustration and anxiety for patients in OAT, particularly when information about the new medication is insufficient or when patients feel coerced to make the change [7, 10]. Although patients expressed trust in treatment staff and reported receiving information from both staff and peers, overall we found that patients perceived information about depot buprenorphine to be insufficient. One possible explanation may be that the information provided might not reflect patients’ experiences at different stages of their treatment. A recent study of patients’ early experiences of treatment highlighted shifting negative and positive states [38] and emphasized the need for staff to inform patients about this and to help them manage their emotions and anxiety during the induction phase. Another factor to consider is the potential cognitive impairment due to ongoing substance use and comorbidities, suggesting that information may need to be adapted and repeated to meet the needs of this unstable patient population.

## Conclusions

In conclusion, this study delves into the experiences of depot buprenorphine treatment among unstable patients with severe polysubstance use and psychiatric comorbidities. While echoing both positive and negative aspects observed in stable and unselected groups of patients, it highlights the potential of depot buprenorphine in facilitating identity change, decreasing diversion to the illicit market, and enhancing treatment retention. However, challenges such as insufficient medication effects and inadequate information dissemination warrant careful consideration, emphasizing the importance of individualized treatment options and targeted communication for this patient population.

### Electronic supplementary material

Below is the link to the electronic supplementary material.

Supplementary Material 1

## Data Availability

Permission to share data is controlled by the ethics permission. Queries regarding the permission to obtain data can be made to the Swedish Ethics Authority, +46 10 475 0800 (email: registrator@etikprovning.se).

## Abbreviations

OAT: Opioid agonist treatment
OUD: Opioid use disorder
